# The taste response to ammonia in *Drosophila*

**DOI:** 10.1038/srep43754

**Published:** 2017-03-06

**Authors:** R. Delventhal, K. Menuz, R. Joseph, J. Park, J. S. Sun, J. R. Carlson

**Affiliations:** 1 Dept. of Molecular, Cellular, and Developmental Biology, Yale University, P.O. Box 208103 New Haven, CT 06520-8103, USA.

## Abstract

Ammonia is both a building block and a breakdown product of amino acids and is found widely in the environment. The odor of ammonia is attractive to many insects, including insect vectors of disease. The olfactory response of *Drosophila* to ammonia has been studied in some detail, but the taste response has received remarkably little attention. Here, we show that ammonia is a taste cue for *Drosophila*. Nearly all sensilla of the major taste organ of the *Drosophila* head house a neuron that responds to neutral solutions of ammonia. Ammonia is toxic at high levels to many organisms, and we find that it has a negative valence in two paradigms of taste behavior, one operating over hours and the other over seconds. Physiological and behavioral responses to ammonia depend at least in part on *Gr66a*^+^ bitter-sensing taste neurons, which activate a circuit that deters feeding. The Amt transporter, a critical component of olfactory responses to ammonia, is widely expressed in taste neurons but is not required for taste responses. This work establishes ammonia as an ecologically important taste cue in *Drosophila*, and shows that it can activate circuits that promote opposite behavioral outcomes via different sensory systems.

Ammonia plays a critical role in biological systems. In the early Earth, ammonia likely combined with water, methane, and hydrogen in the presence of lightning bolts to produce simple organic compounds essential to the first forms of life^1^. Ammonia is an essential building block of amino acids, and it is a breakdown product of amino acids. It is found widely in the environment: in air, in waters, in soils, and in all forms of life^2^.

Ammonia is an olfactory cue for many organisms, including insect vectors of human disease. *Anopheles* mosquitoes that transmit malaria are attracted to ammonia^3^,^4^, which is a component of human sweat^5^, and ammonia is an effective attractant in *Anopheles* traps^6^. *Aedes* mosquitoes, which transmit Zika, dengue fever and yellow fever, are also attracted to the odor of ammonia^7^, as are insects that transmit Chagas disease^8^. Ammonia also attracts the widely used genetic model organism *Drosophila* and several other insect species^9^,^10^,^11^,^12^,^13^, perhaps because ammonia odor indicates protein-rich food sources^10^. The molecular and cellular mechanisms underlying the olfactory response to ammonia have been investigated in the antenna of the fly and the mosquito^14^,^15^,^16^,^17^.

The taste response to ammonia, by contrast, is largely unexplored. In principle, the ammonia in human sweat might elicit an appetitive response via the taste organs of mosquitoes. On the other hand, high levels of ammonia are toxic to most organisms^18^,^19^,^20^, so ammonia might elicit an aversive taste response. There is evidence that mice avoid ingestion of high concentrations of ammonium chloride^21^, and ammonium chloride has a bitter and salty taste to humans^22^,^23^. However, there has been remarkably little study of the taste response to ammonia in insects.

The major taste organ of the *Drosophila* head is the labellum, which contains 31 taste sensilla^24^. These sensilla divide into three morphological classes: long (L), intermediate (I), and short (S)^25^,^26^. Each individual sensillum is identified by its position on the labellum, *e.g.* L_1_. When a sensillum makes contact with a food source, tastants diffuse through a pore in the tip of the sensillum and activate neurons within^24^. For example, some taste neurons are activated by sugars and promote an appetitive response, while other neurons are activated by bitter compounds and elicit an aversive response. L sensilla exhibit strong electrophysiological responses to sugars^27^, but not to bitter compounds; I and S sensilla show strong responses to bitter compounds^26^. The legs, pharynx, and wings also contain taste neurons^24^,^28^,^29^,^30^.

Here we ask whether ammonia elicits a taste response from *Drosophila*. We find that ammonia elicits a strong physiological response from one class of labellar taste sensilla, the S sensilla, and weaker responses from others. Whereas ammonia elicits an attractive response via the olfactory system, we find that it elicits an aversive behavioral response in two taste paradigms. We then explore the cellular and molecular basis of the taste response. We find that physiological responses of the S sensilla and behavioral responses both depend on bitter-sensing neurons that activate an aversive circuit. We then demonstrate that Amt, an essential molecular component of ammonia response in the olfactory system, is also expressed in the taste system, although in a different cell type. Surprisingly, while Amt is required for the olfactory response to ammonia, it is not required for the taste response, indicating differences in the underpinnings of ammonia detection in olfaction and taste.

## Results

### Taste sensilla respond to ammonia

We systematically tested all 31 of the taste sensilla on the labellum for response to ammonia. We used electrophysiological tip recordings to determine whether a solution of ammonium chloride (NH_4_Cl) at neutral pH elicited action potentials from neurons in any of the sensilla. We initially tested NH_4_Cl at a concentration of 100 mM, which is well within the range found in ammonia-rich sources in natural environments^31^,^32^. We note that ammonia exists primarily in gaseous form at room temperature, but dissolves readily in water. At pH 7 most ammonia molecules are protonated, *i.e.* NH_4_^+^, but for simplicity we will refer to both NH_3_ and NH_4_^+^ as “ammonia”.

**Figure 1:**
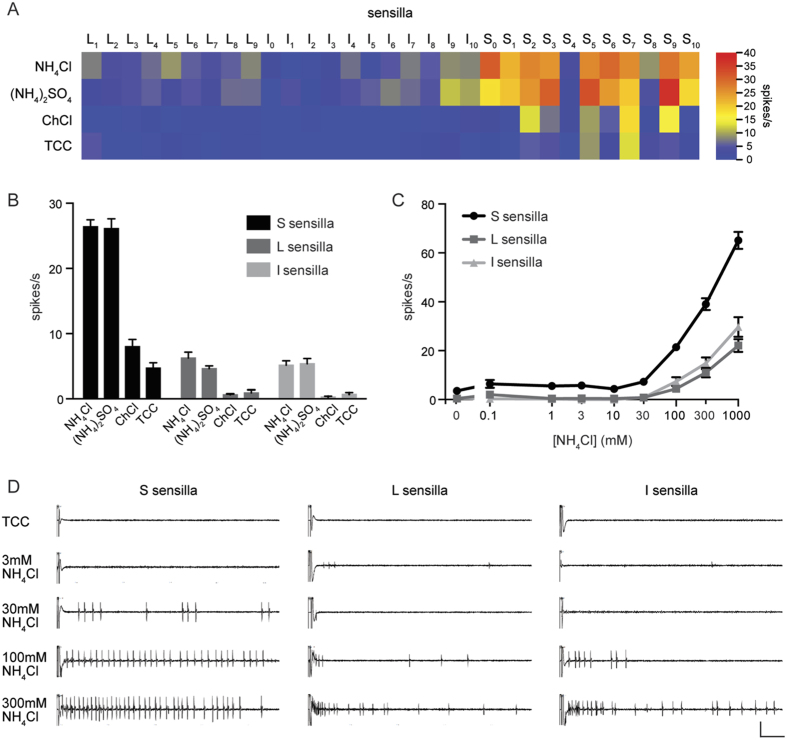
Labellar taste sensilla respond to ammonia. (**A**) Mean responses in spikes/s per individual sensillum type. Responses to the diluent, TCC, along with responses to NH_4_Cl, NH_4_ sulfate, and ChCl are displayed. Values for the diluent were not subtracted from values for the other compounds. NH_4_Cl and NH_4_ sulfate solutions were neutralized to pH 7 with NH_4_OH. n values are provided in Table S1. (**B**) Mean responses of each morphological class of sensilla. S_4_ and S_8_ sensilla were excluded. n = 44–58 (**C**) Taste sensilla respond to ammonia in a dose-dependent manner. “0 mM NH_4_Cl” indicates the response to TCC alone. n = 8–52.(**D**) Representative electrophysiological traces. Scale bar indicates 1 mV, 100 ms.

This NH_4_Cl stimulus elicited strong responses from most S sensilla, and weaker responses from some L and I sensilla (Fig. 1A). We tested two additional stimuli on the same sensilla to determine if the NH_4_Cl responses are in fact due to ammonia, as opposed to the chloride ion. We found that applying an equivalent concentration of ammonia using NH_4_ sulfate, *i.e.* (NH_4_)_2_SO_4_, at pH 7 also elicited strong responses from most S sensilla and weaker responses from L and I sensilla. In contrast, 100 mM choline chloride (ChCl), which contains chloride but not ammonia, elicited weaker responses than NH_4_Cl from all sensilla, indicating that the neurons do not respond to chloride ions. The diluent alone, a solution of tricholine citrate (TCC)^33^, produced little if any response from most of these sensilla (Fig. 1A).

When the responses were grouped by morphological type (S, L, I), each type gave stronger responses to NH_4_Cl or NH_4_ sulfate than to the control stimuli (Fig. 1B; p < 0.02). Moreover, in each sensillum type the responses to NH_4_Cl and NH_4_ sulfate were indistinguishable (p > 0.05). The simplest interpretation of these results is that labellar taste sensilla respond to ammonia.

We note that S_4_ and S_8_, which differ from other S sensilla in their lack of response to bitter compounds^26^, also differ in their lack of strong responses to NH_4_Cl or NH_4_ sulfate (Fig. 1A). Accordingly, S_4_ and S_8_ were excluded from the analysis shown in Fig. 1B and from further analysis of S sensilla.

The responses to ammonia were confirmed and extended by testing NH_4_Cl across a range of concentrations spanning four orders of magnitude (Fig. 1C). Responses were dose-dependent, with response thresholds between 10 mM and 100 mM for each sensillum type. Response magnitudes of S sensilla were higher than those of L or I sensilla at all concentrations above the threshold. Representative traces of responses to increasing NH_4_Cl concentrations are shown in Fig. 1D.

### Ammonia is an aversive taste cue

What is the valence of ammonia taste? Ammonia odor is attractive to many insects, perhaps serving as a cue for protein sources^10^,^34^. However, high concentrations of ammonia are toxic, suggesting the need for a mechanism to avoid their ingestion.

**Figure 2:**
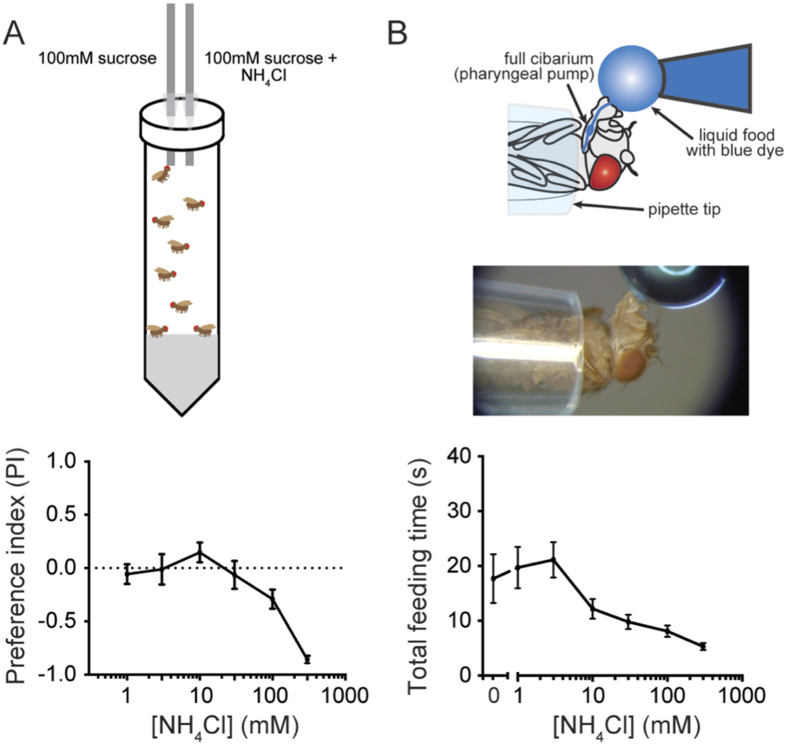
Ammonia is behaviorally aversive. (**A**) Top: CAFÉ assay. Starved flies are housed in a conical tube for 4 h with access to two capillary tubes, one containing 100 mM sucrose alone and the other containing 100 mM sucrose and concentrations of NH_4_Cl ranging from 1 mM to 300 mM. The amount of solution removed from each tube is measured and a preference index (PI) is calculated; a negative PI indicates a preference for sucrose alone. Bottom: flies prefer solutions of sucrose alone to sucrose solutions containing high concentrations of NH_4_Cl (n = 12–28). (**B**) Top: pharyngeal pumping assay. A fly restrained in a pipette tip is presented with a drop of a solution. The time spent feeding from the drop is measured. Bottom: addition of increasing concentrations of NH_4_Cl reduced the time spent feeding (n = 20).

To examine the valence of ammonia taste we used two distinct behavioral paradigms. First we used a CAFÉ assay in which small groups of starved flies were given 4 hours to feed from either of two capillary tubes, one containing sucrose alone and the other containing sucrose mixed with NH_4_Cl^35^. We found that flies preferred solutions containing sucrose alone to those containing sucrose and NH_4_Cl at levels greater than 30 mM (Fig. 2A).

Next we examined feeding over a much shorter time span, using a pharyngeal pumping assay^36^. Starved flies were presented with a water droplet, and the duration of ingestion was measured by visual inspection. Adding 10 mM NH_4_Cl or more to the droplet decreased feeding time, with greater doses producing shorter feeding periods (Fig. 2B).

In summary, NH_4_Cl had a negative valence in two taste paradigms, one extending over hours and the other over seconds. To confirm our interpretation of these results and to extend our analysis of ammonia taste response, we next investigated its cellular basis.

### Taste response to ammonia requires *Gr66a* ^+^ neurons

Which neurons are required in gustatory sensilla for the ammonia response? Previous work has supported a model in which S and I sensilla, but not L sensilla, contain a neuron that expresses the bitter receptor Gr66a, responds to bitter compounds, and activates an aversion circuit^25^,^26^,^37^,^38^. Since ammonia appeared aversive in two behavioral paradigms, we hypothesized that *Gr66a*-expressing “bitter” neurons functioned in ammonia response. To test this hypothesis we ablated bitter neurons by using a *Gr66a-GAL4* construct to drive expression of diptheria toxin (*UAS-DTA*) and then measured neuronal spiking responses to ammonia. To confirm the ablation we also measured responses to berberine, a well-characterized bitter compound.

**Figure 3:**
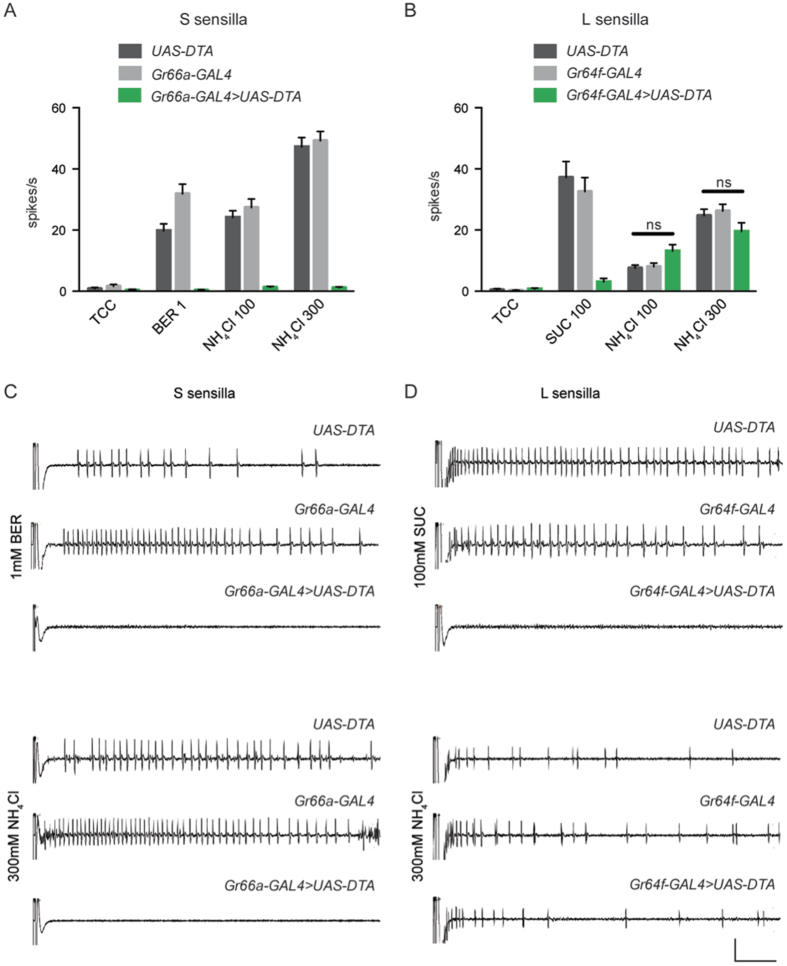
Ammonia responses depend on defined neurons. (**A**) Ablation of bitter-sensitive *Gr66a*^+^ neurons with a *Gr66a-GAL4* driver and *UAS-DTA* (diphtheria toxin) severely reduces responses of S sensilla to the bitter compound berberine (1 mM) and to NH_4_Cl (n = 22–37, p < 0.0001). Parental control lines respond strongly to these stimuli. (**B**) Ablation of sugar-sensitive *Gr64f*^+^ cells severely reduces the response to 100 mM sucrose in L sensilla (p < 0.0001), but does not affect responses to NH_4_Cl (n = 22–27). (**C**,**D**) Representative electrophysiological traces. Scale bar indicates 1 mV, 100 ms.

Physiological analysis of S sensilla from control flies containing either *Gr66a-GAL4* or *UAS-DTA* alone produced strong responses to both berberine and NH_4_Cl (Fig. 3A,C). However, S sensilla of flies containing both constructs showed virtually no response to either berberine or NH_4_Cl. The same conclusion was reached with each of two concentrations of NH_4_Cl. The simplest interpretation of these results is that the ammonia responses of S sensilla require the *Gr66a*^+^ neuron.

We also ablated neurons that express the sugar receptor *Gr64f*, respond to sugars, and activate an appetitive response^27^,^39^. For this experiment we examined L sensilla, whose sucrose responses are much more robust and consistent in our hands than those of S sensilla. Physiological analysis of sensilla from control flies containing *Gr64f-GAL4* alone, or *UAS-DTA* alone, produced strong responses to sucrose (Fig. 3B,D). However, sensilla of flies containing both constructs showed a dramatically reduced response to sucrose, confirming successful ablation of the sugar neuron. Responses to NH_4_Cl were the same in all three genotypes, at each of two different NH_4_Cl concentrations. The simplest interpretation of these results is that these ammonia responses do not depend on sugar neurons.

**Figure 4:**
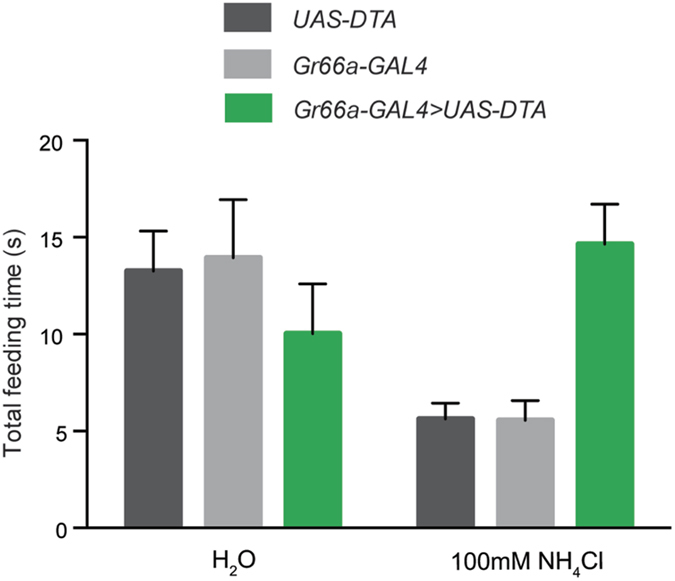
Aversion to ammonia depends on *Gr66a*^+^ neurons in the pharyngeal pumping paradigm. Flies in which *Gr66a*^+^ bitter-sensitive neurons were ablated with *Gr66a-GAL4* and *UAS-DTA* spent the same amount of time feeding on a water droplet as parental controls. Flies in which *Gr66a*^+^ cells were ablated spent longer feeding on 100 mM NH_4_Cl than parental controls (p < 0.001; one-way ANOVA, Bonferroni post-test, n = 26).

Behavioral analysis was carried out to determine whether aversion of the fly to ammonia depended on *Gr66a*^+^ taste neurons. We used the pharyngeal pumping paradigm, which measures the response to NH_4_Cl and does not entail the use of sucrose, as does the CAFÉ assay. We found that in the absence of NH_4_Cl, feeding time on a water droplet was equivalent in flies containing *Gr66a-GAL4* alone, *UAS-DTA* alone, or *Gr66a-GAL4; UAS-DTA* (Fig. 4). However, in the presence of NH_4_Cl, flies in which *Gr66a*^+^ cells were ablated showed increased feeding time compared to the parental controls. These results indicate that bitter neurons are required for the aversive, anti-feedant response to NH_4_Cl.

### The molecular underpinnings of ammonia response differ between taste and olfaction

We recently identified in *Drosophila* an ammonia transporter, Amt, which is expressed in a class of olfactory sensilla that respond strongly to airborne ammonia^15^. Amt is expressed in auxiliary cells of these sensilla. Analysis of an insertion mutation revealed a requirement for Amt in the electrophysiological response of these olfactory sensilla to ammonia.

**Figure 5:**
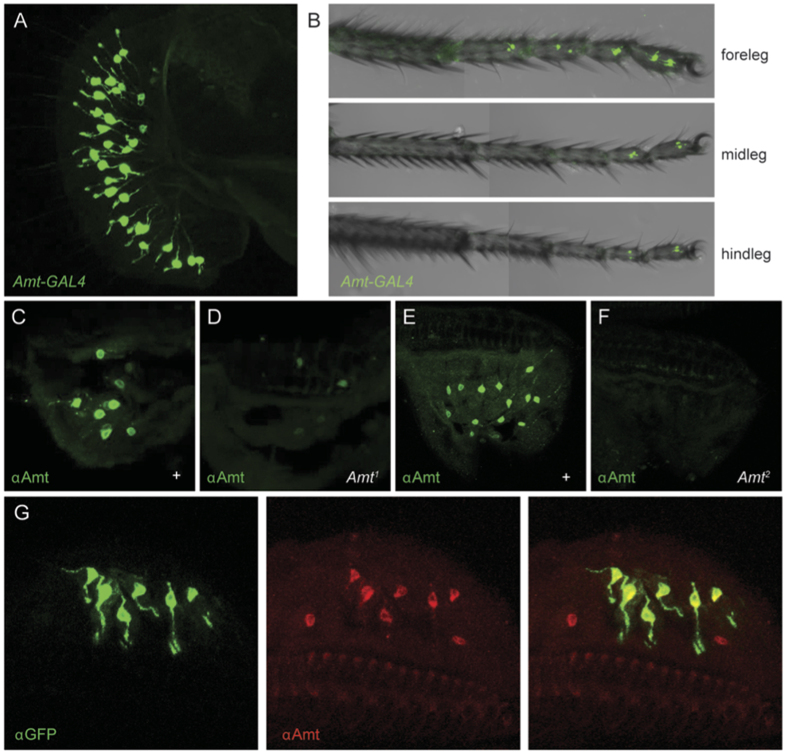
The ammonia transporter Amt is expressed in gustatory neurons in the labellum. *Amt-GAL4* drives *UAS-mCD8::GFP* in taste neurons in (**A**) the labellum and (**B**) legs, as visualized in whole mount preparations. (**C**) An αAMT antibody labels cells in labellar sections of *w*^*iso*^ control flies, but (**D**) most staining is lost in *Amt*^*1*^. (**E**,**F**) Labeling is eliminated in the deletion allele *Amt*^*2*^ compared to the control *wCS*. (**G**) In *Amt*^+^ flies, all neurons that express GFP driven by *Amt-GAL4* are also labeled by the αAMT antibody. The αAMT antibody also labels some cells that show weak or no labeling with *Amt-GAL4*.

We found that an *Amt-GAL4* construct drives expression of a *UAS-GFP* reporter in two taste organs: the labellum and legs (Fig. 5A,B), in addition to the antenna. Surprisingly, in these taste organs Amt expression was detected in neurons, identified by the presence of labeled dendrites and axons, rather than in auxiliary cells. To confirm the expression of Amt in taste neurons, we generated an antibody to the 148 amino acids of the C-terminal tail of Amt. This antibody labeled neurons in taste sensilla of the labellum, confirming the results obtained with the *Amt-GAL4* driver (Fig. 5C). The specificity of the antibody was confirmed by testing it against the *Amt*^*1*^ insertion mutant: labeling was greatly reduced in the labellum (Fig. 5D). Labeling was not completely eliminated, however, presumably reflecting the presence of some residual Amt expression in the insertion mutant.

Using CRISPR-Cas9 technology we generated *Amt*^*2*^ 40, in which most of the protein-coding sequence of the gene, including 8 of 11 transmembrane domains, are deleted (Fig. S1). The mutation was outcrossed against a control *wCS* stock for five generations. The *Amt*^*2*^ labellum revealed no labeling with the anti-Amt antibody, providing further confirmation of the antibody’s specificity (Fig. 5E,F). In *Amt*^+^ animals, all neurons labeled by *Amt-GAL4; UAS-GFP* were also labeled by the antibody (Fig. 5G), as expected of a faithful *GAL4* driver; a small fraction of neurons labeled with the antibody were labeled weakly if at all by the driver, as often found for drivers that are not expressed as strongly as the genes they represent.

*Amt-GAL4* is expressed in a single neuron per sensillum on the labellum. All or nearly all sensilla contain a neuron that expresses the driver (Fig. 5A).

**Figure 6:**
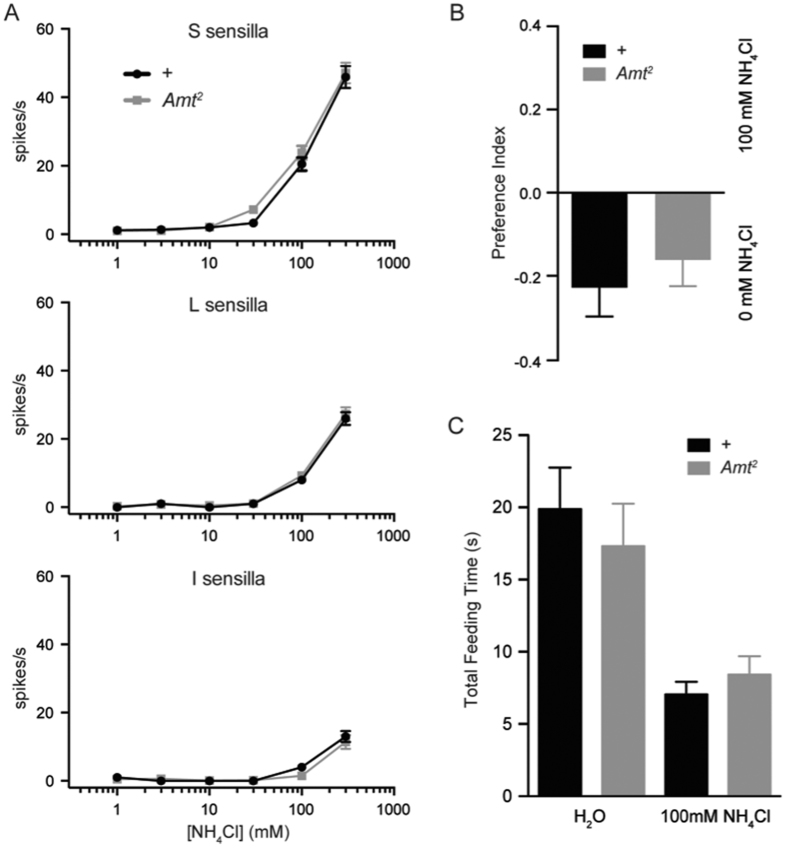
*Amt* is not required for taste responses to ammonia. (**A**) *Amt*^*2*^ flies have electrophysiological responses to NH_4_Cl that are not significantly different from responses of *wCS* controls (+). (**B**) *Amt*^*2*^ flies show the same preference for sucrose alone (0 mM NH_4_Cl) versus sucrose with 100 mM NH_4_Cl as controls in the CAFÉ assay (n = 47–57). (**C**) In the pharyngeal pumping assay, the time spent feeding on 100 mM NH_4_Cl by *Amt*^*2*^ flies is not significantly different from *wCS* controls (+) (n = 25–31).

Is *Amt* required for gustatory response to ammonia, as it is for olfactory response? We were surprised to find that *Amt*^*2*^ showed electrophysiological responses similar to those of the control line across a broad concentration range, in S, L, and I sensilla (Fig. 6A). *Amt*^*2*^ also showed a normal behavioral response in a CAFÉ assay to 100 mM NH_4_Cl, a concentration that elicits an intermediate response in wild type flies (Fig. 6B). Finally, *Amt*^*2*^ had a normal response in the pharyngeal pumping paradigm when tested with either water or ammonia (Fig. 6C).

These results prompted us to map *Amt* expression in more detail. We carried out double-label experiments using *Amt*-*GAL4* and markers of sugar (*Gr5a-LexA*) or bitter (*Gr66a-RFP*) neurons. These experiments revealed expression of *Amt* in sugar neurons, but not bitter neurons (Fig. S2). This mapping is consistent with our finding that neither loss of *Amt* nor ablation of sugar cells affects ammonia response (Fig. 3).

## Discussion

Ammonia has long been known to act as an olfactory cue for many animals^7^,^8^,^9^,^10^,^11^,^12^,^41^,^42^,^43^. Here we show that it also serves as a taste cue for the fly.

### Ecological function of ammonia taste response

Why have flies evolved a taste response to ammonia? The taste response to sugars identifies sources of nutrition. The taste response to bitter compounds signals the presence of a wide diversity of toxic molecules that are produced by plants^44^. Ammonia, an inorganic compound that is chemically much simpler, is also toxic to animals at high concentrations^18^,^19^,^20^. The concentration of ammonia in fly culture vials may approach 30 mM^45^, near the threshold of the physiological responses we have found for ammonia. It seems likely that the taste response to ammonia thus warns the fly of the toxicity of food sources with high ammonia levels and inhibits their consumption. In support of this interpretation, we found that high levels of ammonia inhibit consumption in each of two feeding paradigms, one carried out over the course of hours and the other over seconds. We note that the threshold for ammonia detection appears somewhat higher in the CAFÉ assay than in the pharyngeal pumping assay, perhaps because of the presence of sucrose in the CAFÉ assay.

### Opposite valences in taste and olfaction

The negative valence of ammonia as a taste cue is in striking contrast to its positive valence as an olfactory cue. Ammonia is aversive in our taste paradigms, yet is attractive in olfactory paradigms for either *Drosophila* or insect vectors that seek human hosts on which to feed^3^,^7^,^8^,^9^. Ammonia can thus activate sensory circuits that promote opposite outcomes.

There is precedent for compounds that activate opposing responses via the same sensory system, when presented at different concentrations. For example, low concentrations of the odorant ethyl acetate are attractive in an olfactory paradigm, while high concentrations are repellent^46^. Likewise, low concentrations of the tastant NaCl are appetitive in taste paradigms, while high concentrations are aversive^25^.

There is also precedent for compounds that activate both olfactory and gustatory circuits. The insect repellent DEET elicits responses from both systems, but in each case the response is of the same valence^47^. In *Drosophila*, CO_2_ activates the olfactory and gustatory systems with different valences^48^,^49^. However, the case of CO_2_ is distinct from that of ammonia, in that CO_2_ is aversive to flies when presented as an olfactory stimulus, but is attractive when presented as a gustatory stimulus. In contrast, recent work suggests that like ammonia, the odor of polyamines is attractive, whereas their taste is aversive^50^.

The ability to sense ammonia via two sensory systems and to activate circuits promoting opposing outcomes may be highly beneficial to insects in their natural environments. Low concentrations of ammonia detected via olfaction may signal the availability of a food source at a distance, whereas high concentrations sensed via gustation warn of toxicity and may signify that local nutrients have been metabolized.

Our work invites a detailed analysis of the taste responses of insect vectors of human disease to ammonia. Human sweat contains on the order of 2 mM–5 mM ammonia^5^, which is below the thresholds of physiological or behavioral taste response that we have found for *Drosophila*. It will be interesting to determine whether these levels activate an appetitive taste circuit in insects that are attracted to humans, land on them, and make feeding decisions after landing. We note the possibility of a synergistic effect between ammonia and other sweat compounds in eliciting taste responses; such synergy is observed in eliciting olfactory responses^4^,^7^.

### The cellular basis of ammonia taste

We found that ammonia is detected by taste sensilla on the labellum, with S sensilla responding strongly and L and I sensilla responding less strongly. Ablation experiments showed that the physiological responses of S sensilla depend on the Gr66a^+^ bitter-sensing neuron. Likewise, aversion to ammonia in a feeding assay depended on bitter-sensing neurons. These results are consistent with previous findings that these neurons activate an aversive response to bitter compounds. The results suggest that ammonia activates an aversive circuit that is also activated by plant-derived bitter compounds.

L sensilla do not contain a canonical bitter-sensing neuron^25^,^26^, so which neuron mediates their ammonia response? The response does not derive from the sugar-sensing neuron, since ablation of this neuron in L sensilla did not reduce the physiological response to ammonia. In S and I sensilla, the bitter-sensitive neuron also responds to high concentrations of salt, an aversive stimulus^25^. In L sensilla, one of the four neurons is also responsive to high salt^25^. It seems plausible that the high-salt sensing neuron in L sensilla also senses ammonia, as in S sensilla. However, testing this hypothesis will require a suitable means of manipulating this neuron specifically.

### The molecular basis of ammonia detection differs between olfaction and taste

We were surprised to find that the ammonium transporter Amt, which is required for olfactory response to ammonia^15^, is not required for gustatory response to ammonia, despite its expression in taste sensilla. Also unexpected was the localization of Amt to sugar-sensing neurons of taste sensilla, as opposed to support cells, as in olfactory sensilla.

Previous findings are also consistent with differences in the molecular basis of ammonia detection in olfaction and taste. In the antenna, strong ammonia responses are mediated by the neuronal receptor IR92a, which was not detected in the labellum^9^,^51^. Given that other IR family members are expressed in the labellum, another IR family member could mediate ammonia detection in this organ. At least one IR is expressed in some bitter-sensing neurons of the labellum^52^, and the labellar expression of several other IRs has not yet been examined.

One interpretation of these differences is that the underlying mechanisms of ammonia detection differ between olfaction and taste. In olfaction, low concentrations of airborne ammonia enter the sensillum via openings in the sensillar walls, and are then detected by olfactory neurons expressing IR92a. The basal ammonia concentration of the sensillum lymph is unknown, but the concentration in the larval hemolymph is ~1 mM^53^, and it is possible that the transport uptake function of Amt is needed to keep the ammonia concentration in the lymph of an olfactory sensillum sufficiently low to allow detection of low levels of airborne ammonia. By contrast, the ammonia levels that activate taste neurons (10–100 mM) and those found in a fly culture vial (~30 mM)^45^ are much higher than 1 mM. Thus the need to keep the ammonia concentration very low in the lymph of a taste sensillum may be less acute. In any case this unexpected difference invites a detailed investigation of the molecular mechanisms of ammonia taste.

In summary, in this study we have found that ammonia elicits a taste response from *Drosophila*, and we have characterized this response at the physiological, cellular and behavioral levels. We have identified taste sensilla that detect ammonia and have characterized their electrophysiological responses. We have shown that ammonia is an aversive stimulus in feeding assays, and that both physiological and behavioral responses depend on a class of bitter-sensitive neurons. Our results suggest a means by which flies may avoid ingesting toxic levels of ammonia, but remain attracted to low levels of airborne ammonia. These results lay a foundation for a molecular investigation of the mechanism of ammonia taste.

## Materials and Methods

### *Drosophila* stocks

Flies were grown on standard cornmeal-agar medium at 25 °C in a humidity-controlled incubator. The control flies used in Fig. 1 contained a *piggyBac* transposon, *pBacWHf04393*, that is not expected to affect ammonia responses. *wCS* flies were used for behavioral assays, unless otherwise noted. *Gr66a-GAL4*^26^, *Gr64f-Gal4*^27^, and *UAS-DTA* (Bloomington Stock 25039) flies were outcrossed into *wCS* for 5 generations before electrophysiological and behavioral assays. *Amt-GAL4; UAS-mCD8::GFP, Amt*^*1*^, and the *Amt*^*1*^ control, an isogenized *w*^*1118*^ line, were described previously^15^. *Amt*^*2*^ was generated as described below and was compared to its genetic control line, *wCS. Gr5a-LexA*^54^ was obtained from Kristin Scott. *LexAop-m-tdTomato*^52^ and *Gr66a-RFP*^27^ were previously generated by the Carlson lab.

### Generation of the Amt^2^ allele

CRISPR-Cas9 genome engineering with homology-directed repair was used to generate the *Amt*^*2*^ null allele. This mutation eliminates ~63% of the *Amt* coding sequence, leaving 409-nt of coding sequence at the 5′ end and 233-nt at the 3′ end. The remaining sequence contains only the first three of eleven transmembrane regions and is likely to be a null allele.

#### Guide chiRNA cloning

Gibson Assembly was used to clone pU6-BbsI-chiRNA^55^,^56^ plasmids containing each of the chiRNAs, following protocols for Gibson Assembly Master Mix (New England BioLabs, Inc., Ipswich, MA). The reverse primer is complementary to the plasmid itself (chiRNA R), whereas the forward primers also include the 20-nt guide chiRNA sequence with the PAM sequence omitted (Amt 5pchiRNA F and Amt 3pchiRNA F) (Table 1). 20-nt guide chiRNAs were selected with the aid of the CRISPR Optimal Target Finder resource on the flyCRISPR website^40^. CRISPR targets with 5′ G and NGG PAM sequences were selected for optimal U6-driven transcription and subsequent Cas9 cleavage stringency. Primer sequences complementary to the plasmid sequence are represented as lowercase.

#### Homology-directed repair template cloning

Homology arms extending 0.98 kb upstream and 1.03 kb downstream of the Cas9 cut site were incorporated into multiple cloning sites of the pHD-DsRed-attP vector^40^ using amplification primers (Table 2).

To facilitate screening of *Amt* deletion mutants, this replacement donor plasmid contains removable *LoxP*-flanked *DsRed*, which was inserted in the genome at the same locus. A simultaneously inserted attP ΦC31 site facilitates future targeting of this locus.

#### Embryo injection

*y*^*2*^ *cho*^*2*^ *v*^*1*^*; attP40*{*nos-Cas9*}/*CyO* (CAS-0001, ref. 57) embryos were injected with guide chiRNA and donor plasmids by Bestgene, Inc. (Chino Hills, CA). G0 adults were crossed to *w*^*1118*^ flies. G1 adults expressing *DsRed* were identified, and were backcrossed to *wCS* for five generations.

### Electrophysiology

Single-sensillum recordings were performed as described in Delventhal *et al*.^58^. Only females, aged 6–8 days, were used for electrophysiological recording. To quantify responses, the number of action potentials (spikes) was counted over a 500 ms period, starting 200 ms after contact. A high salt stimulus (400 mM NaCl) was used as a positive control at the beginning and end of the recording session for each sensillum to ensure that at least one GRN was functional. All recordings from sensilla that displayed an average NaCl response of less than 10 spikes/s at the beginning or end of a recording session were discarded (representing less than 10% of overall recordings performed). No more than eight tastants were tested on an individual sensillum of a given fly, with 2–3 minutes between presentations. Details on mean responses, SEM and n for electrophysiology experiments are provided in Supplementary Table 1.

Ammonia solutions were prepared at the indicated concentrations with either NH_4_Cl or (NH_4_)_2_SO_4_. In Fig. 1A,B, 100 mM NH_4_Cl and 50 mM (NH_4_)_2_SO_4_ were used to compare equivalent levels of ammonia. All tastants were dissolved in 30 mM tricholine citrate (TCC), an electrolyte that inhibits the water neuron^33^. Solutions were then brought to a pH of ~7 with NH_4_OH. Solution aliquots were stored at −20 °C long-term and kept at 4 °C while in use, for no more than a week.

### CAFÉ assay

We used a modified version of the CAFÉ assay, similar to that originally described^35^. The chamber was prepared by filling a 50 ml conical tube with 30 ml of 2% agarose. Two holes were punched into the cap, and shortened 1 ml pipette tips were inserted through the holes partially into the chamber. Calibrated glass capillary tubes (Drummond Scientific Company, Catalog #2–000–001) were filled with liquid food by capillary action and inserted into the chamber through the pipette tips. Two tubes with liquid food were present in each chamber: one with 100 mM sucrose alone and the other with 100 mM sucrose and varying concentrations of NH_4_Cl.

For the assay, 13 female and 2 male flies (7 days old) were introduced into the CAFÉ chamber, and starved overnight in a 25 °C incubator. Two capillary tubes were introduced the next morning, and flies were given four hours to ingest the liquid food. The amount of solution ingested from each tube was measured, and the preference index (PI) value was calculated according to the formula (X − Y)/(X + Y), where X is the amount ingested from the tube containing sucrose alone and Y is the amount ingested from the tube containing sucrose and ammonia. Average values ± SEM are given.

### Pharyngeal pumping assay

We used a pumping assay similar to that described by ref. 36. In brief, we used 7 day old female flies, which were starved 12–14 hours. Each fly was anesthetized and placed in a 1000-μl pipette tip. A second 1000-μl pipette tip was inserted into the first tip, thereby containing the fly for the subsequent starvation period. Flies were then kept in a 100 mm Petri dish with 3 Kimwipes wetted with 5 ml water to prevent dehydration.

After starvation, gentle aspiration was used to immobilize a fly for food presentation, as follows: a pulse of air was applied to the broad opening of the inserted 1000-μl pipette tip, such that the fly was pushed into the narrow opening of the second 1000-μl pipette tip, thereby immobilizing the fly with its head and proboscis exposed. Occasionally, ends of the 1000-μl pipette tips had to be trimmed with a razor blade, to widen the tip so as to accommodate the fly’s head. The prepared fly was then mounted on a micromanipulator for analysis under a Nikon SMZ800 stereomicroscope. Flies were filmed with a Sony HD Camcorder that was attached to the microscope through an adapter.

A drop of liquid medium was delivered from a P20 PipetteMan mounted on a micromanipulator, which allowed for fine adjustments during the delivery process. The liquid drop was carefully presented to the labellum, such that liquid only contacted the sensory hairs and cuticle present on the labellum. The fly was presented liquid medium for 2 seconds, during which it had the chance to respond by extending its proboscis to drink. Flies that began pharyngeal pumping to ingest the solution were allowed to continue until they ended the bout by breaking contact with the liquid drop. After breaking contact with the solution, flies were given 3 seconds rest before being presented the liquid solution once again. They were then given 2 seconds to initiate a second bout. This process was repeated until the fly no longer responded to presented media, after which the experiment was terminated. Typically, 2 seconds was a sufficient presentation time, as longer presentation times did not appreciably increase the likelihood that a fly would initiate a subsequent feeding bout. On average flies engaged in 1–3 bouts.

For flies that did not respond during the initial 2 second presentation, the liquid solution was removed and the fly was given 3 seconds rest before being presented the liquid solution once again. This presentation process was repeated four times. If flies did not initiate a feeding bout after the fourth presentation, they were considered non-responders and discarded. Approximately half of all flies were non-responders in the case of each genotype.

Flies were offered solutions containing varying concentrations of NH_4_Cl. Each fly was used for only one experiment to prevent previous experience from influencing its response decisions. The video was analyzed at a later time, during which the total time spent ingesting the solution was measured using QuickTime Media Player. When different compounds were tested, the analysis was conducted blind to the stimulus. When different genotypes were tested, the analysis was carried out blind to genotype. Average values ± SEM are indicated for responders.

### Whole-mount imaging

Labella and legs were dissected from male and female flies (7–21 days) on a Sylgard plate. Tissues were immersed in a solution of 0.5X PBS, 0.1% Tween-20, and 50% glycerol for at least five minutes. Tissue was mounted on glass slides and imaged within one day. Confocal stacks were acquired on a Zeiss LSM510 confocal microscope and processed using NIH ImageJ (version 1.44o).

### Generation of αAmt antibody

The last 148 amino acids of the Amt cytosolic C-terminal tail were expressed with a 6xHis-tag at the N-terminus in *E. coli* and affinity-purified by Novogene. Polyclonal antibodies against this protein antigen were generated in a guinea pig through standard protocols (Cocalico Biologicals, Inc.). Serum was used for immunofluorescence experiments at a 1:200 dilution.

### Immunofluorescence labeling of labellar sections

7 day old flies were anesthetized, placed in a collar, covered with OCT (Tissue-Tek), and frozen on dry ice. 40 μm labellar sections were collected on slides, and stored at −80 °C up to one week. Slides were briefly thawed before a 10 minute fixation in 4% formaldehyde in PBS. Sections were washed 3 × 5 minutes in PBS, permeablized for 30 min in PBST (PBS plus 0.1% Tween-20) with 0.2% Triton X-100, blocked for 30 minutes in 1% BSA-PBST, then incubated overnight at 4 °C with primary antibodies diluted in 1% BSA-PBST. Anti-Amt was used at 1:200 and mouse anti-GFP (Roche) was used at 1:500. The following day, sections were washed 3 × 10 minutes in PBST, then incubated for 2 hours with Alexa Fluor secondary antibodies diluted in 1% BSA-PBST. Sections were washed 3 × 5 minutes in PBST and mounted in Vectashield. All microscopy was performed using a Zeiss LSM 510 Laser Scanning Confocal Microscope, and images were processed with ImageJ software.

## Additional Information

**How to cite this article**: Delventhal, R. *et al*. The taste response to ammonia in *Drosophila. Sci. Rep.* **7**, 43754; doi: 10.1038/srep43754 (2017).

**Publisher's note:** Springer Nature remains neutral with regard to jurisdictional claims in published maps and institutional affiliations.

## Supplementary Material

Supplementary Information

Supplementary Table S1

## Figures and Tables

**Table 1:** Guide chiRNA cloning primers.

Primer Name	Sequence
chiRNA R	gaagtattgaggaaaacata
Amt 5pchiRNA F	GCAGCAGTGGCAATCCTACCgttttagagctagaaatagc
Amt 3pchiRNA F	GGTTACTCACCGTTCAGCCAgttttagagctagaaatagc

**Table 2:** Homology arm cloning primers.

Primer Name	Sequence	Restriction Enzyme
AmtH1F	GCGCCTgaattcGGGTCACTGTATCTGTTTGTACATATGT	EcoRI
AmtH1R	GCGCCTccgcggCCATGGCGCCGCTGAC	SacII
AmtH2F	GCGCCTactagtACCTGGAGCAGGTGAGTGGTTCG	SpeI
AmtH2R	GCGCCTctcgagCGACAACTGCTCCCCGTAGTTCA	XhoI
