# Supplementary material for: The taste response to ammonia in Drosophila
Source: Sci Rep. 2017 Mar 6;7:43754. doi: 10.1038/srep43754 (PMC5338342; doi:10.1038/srep43754)
Supplement: Supplementary Information [file srep43754-s1.pdf]

The taste response to ammonia in *Drosophila*

SUPPLEMENTARY INFORMATION

Delventhal, R.[#], Menuz, K.[#], Joseph, R., Park, J., Sun, J.S. and J. R. Carlson*

Dept. MCD Biology
Yale University
P.O. Box 208103
New Haven, CT 06520-8103

#equal contributors
*corresponding author: john.carlson@yale.edu

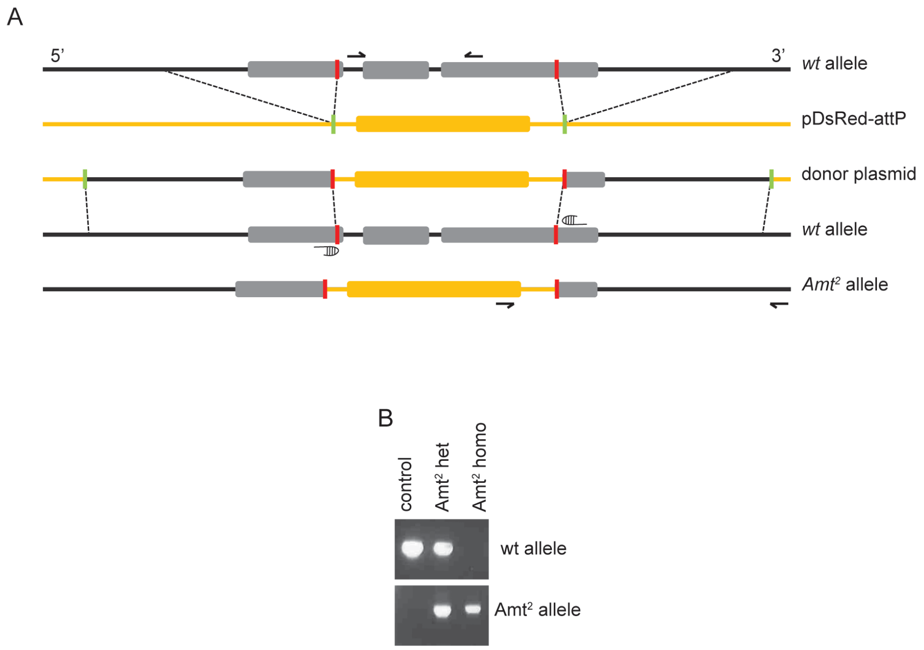

Figure S1. Generation of the *Amt*^{2} allele via CRISPR-Cas9 genome engineering. (A) 5' and 3' homology arms including portions of the first and last exons (gray rectangles) of *Amt* were cloned into the *pHD-DsRed-attP* vector (yellow) to generate the donor plasmid. Introduction of the guide RNAs (small gray RNA symbols) and the donor plasmid into *y*^{2} *cho*^{2} *v*^{1}*; attP40{nos-Cas9}/CyO* flies with a wildtype *Amt* allele (wt) led to the generation of the *Amt*^{2} allele via homology-directed repair. Mutant flies can be identified by expression of DsRed or by PCR using primers specific for the wt and *Amt*^{2} alleles (black arrows on wt and *Amt*^{2} alleles, respectively). (B) PCR analysis with genomic DNA identifies the wt allele in control and *Amt*^{2}*/+* heterozygous flies, and the *Amt*^{2} mutant allele in heterozygous and homozygous mutant flies.

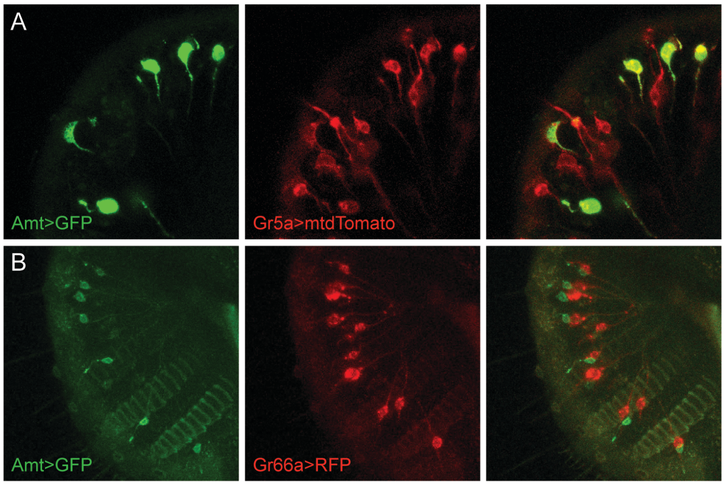

Figure S2. Amt is expressed in sugar-sensitive neurons. (A) Whole mount labella prepared from *Gr5a-LexA; Amt-GAL4; UAS-mCD8::GFP, LexAop-m-tdTomato* flies. Amt is expressed in a subset of sugar-sensitive *Gr5a*$^{+}$ neurons. (B) In contrast, Amt is not expressed in bitter-sensitive *Gr66a*$^{+}$ neurons, as seen in *Amt-GAL4/Gr66a-RFP; UAS-mCD8::GFP/Gr66a-RFP* flies.

(Please see separately uploaded Excel document)

Table S1. Values for electrophysiological recordings
(Figure 1A,B) Mean spikes/sec, SEM and n by sensillum type. In general, we recorded from 5-9 sensilla for each tastant for sensilla that respond strongly to ammonia (>10 spikes/s), and 2-9 sensilla for sensilla that responded weakly. As shown in the Table, fewer sensilla were recorded for I10 and S0, which are particularly difficult to access for recordings due to their location.
(Figure 1C) Mean spikes/sec, SEM and n by NH_4Cl concentration in mM.
(Figure 3A) Mean spikes/sec, SEM, and n by tastant and genotype for S type sensilla.
(Figure 3B) Mean spikes/sec, SEM, and n by tastant and genotype for L type sensilla.
(Figure 6A) Mean spikes/sec, SEM, and n by sensilla, tastant and genotype.
